# Temporal Lobe Parenchyma Herniation into the Transverse Sinus: MRI Findings in a Case

**DOI:** 10.5334/jbr-btr.1001

**Published:** 2016-01-29

**Authors:** Elçin Aydin, Hasan Yerli, Esin Gezmiş

**Affiliations:** 1 Başkent University, TR

**Keywords:** brain, herniation, MRI, venous sinus, headache

## Abstract

Brain parenchyma herniation into dural venous sinus which is a uncommon entity, can cause dural venous sinus filling and simulate sinus thrombosis and other pathologies. It is isointense to brain parenchyma on all sequences by magnetic resonance imaging, surrounded by a cerebrospinal fluid rim and is seen to be contiguous with brain tissue on images. We report a rare case with spontaneous occult herniation of temporal lobe tissue into the left transverse sinus that may associated with headache.

There are several causes of dural venous sinus filling defects including arachnoid granulations, sinus thrombosis, tumours, intrasinus septa (fibrotic bands) and hypoplasia or aplasia of dural sinuses. Brain herniation with surrounding cerebrospinal fluid (CSF) into the dural venous sinuses is a uncommon entity and can simulate aforementioned pathologies and variations causing dural venous sinus filling. It was recently described on magnetic resonance imaging (MRI) and it is also named as ‘‘encephalocele” and “invagination”. Although its clinical significance is controversial, it is suggested that brain herniations may cause some symptoms suchs as headache, dizziness, syncope and imbalance. We report a rare case with headache and spontaneous occult herniation of temporal lobe tissue into the left transverse sinus.

## Case report

**Figure 1:**
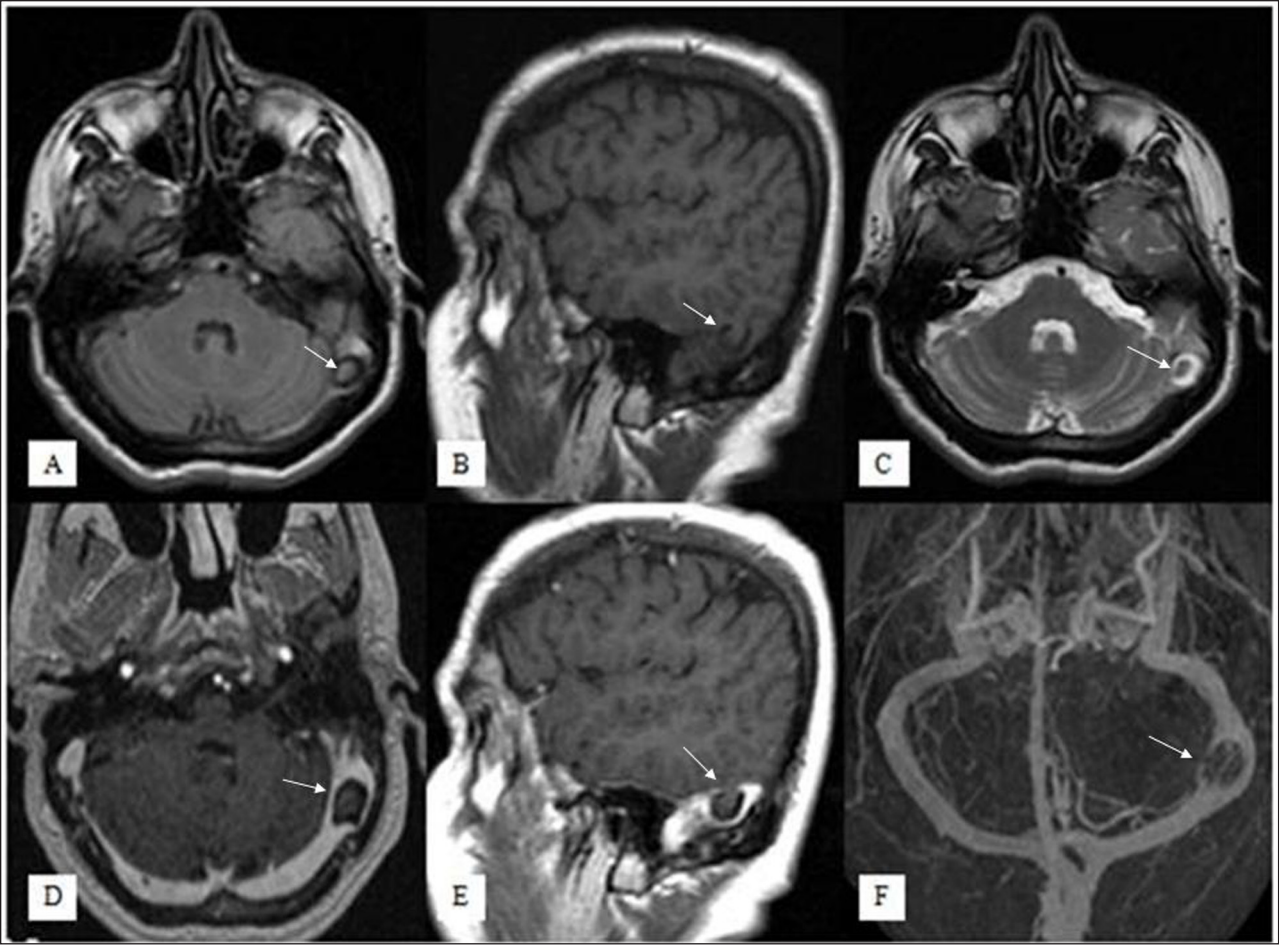
Fluid Attenuated Inversion Recovery axial (A), T1-weighted sagittal (B), T2-weighted axial (C), contrast-enhanced T1-weighted axial (D) and sagittal (E) images show a small herniation of temporal lobe parenchyma with surrounding CSF into left transverse sinus, that was isointense to brain parenchyma on all sequences (arrows). No pathological enhancement is seen but the brain herniation sac is causing moderate stenosis in the left transverse sinus. On venography imaging (F), there was left transverse sinus stenosis but no venous thrombosis.

A-50-year-old female presented with history of headache for a long time. Her neurological examination was normal. The laboratory results were within normal limits. Brain MRI demostrated a small herniation of a temporal lobe with surrounding CSF rim into the left transverse sinus (Figures 1A–E, arrows). The herniation material was isointense to brain parenchyma on all sequences in the contiguous brain tissue images. T2-weighted axial image showed loss of signal void in the left transverse sinus (Figure 1C). After contrast media administration, no pathological parenchymal or meningeal opacification was seen, the herniation tissue was seen to bulge into left transverse sinus and it was caused the narrowing of the sinus (Figure 1D,E). On MR venography imaging, there was left transverse sinus stenosis but no venous thrombosis (Figure 1F).

## Discussion

Brain parenchyma herniations into a dural venous sinus are rare entities which can be seen as filling defects of dural sinuses on radiological images [1]. Brain parenchyma herniations are different from classic encephaloceles that composed of meninges and brain located outside of the skull. Suggested main mechanisms regarding with classic encephalocele in the literature are non-union of ossification centres in bones or variations of bone thickness that may cause brain tissue herniation by pressure of the brain tissue or CSF [2]. Calvarial defects can be seen in radiological images in the form of classical herniation in the skull level. Conversely, there is no bone defect in the brain parenchyma herniation unlike the classical encephalocele. The mechanism of brain parenchyma herniations as it is in the classic encephaloceles is not clear. Progressive dural thinning secondary to elevated CSF pressure, inflammation, aging, and erosive arachnoid granulations are among the etiologies thought to be responsible [3].

Classical encephalocele can sometimes cause CSF leakage, epilepsy, meningitis and ear disturbances such as hearing loss, otorrhea and otitis media [3]. On the other hand, brain herniation into dural sinuses may associated with some different symptoms such as headache, syncope, dizziness and imbalance although the relationship between herniated brain and symptoms are indefinite [3]. Our patient was complaining about headache. In literature, it is reported some cases with headache and brain herniation into dural sinuses. Battal et al. described four brain herniations into transvers sinuses and they observed that two of them had histories of headaches [4]. Karatag et al. describe a case of temporal lob herniation into the sigmoid sinus which had a history of headache [1]. The tension of duramater and vessels due to the pressure of the herniation may cause headache but there is not enough information in the literature and exact pathogenesis is unclear. Long-term follow-up studies are needed to be understood if symptoms are in relation with radiological findings or not.

The main differential diagnosis for the lesions causing dural venous sinus filling defect includes dural sinus thrombosis, arachnoid granulations and tumor. The MR signals depends on clot age in the dural sinus thrombosis. Acute thrombosis is seen as isointense, hypointense and hyperintense on T1, T2 and T2*-weighted images, respectively. Subacute sinus thrombosis shows hyperintense signals on T1, T2 and T2*-weighted images. In the chronic stage, thrombosis shows isointense signals on T1-weigted images and moderately hyperintense signals on T2-weighted images and postcontrast T1-weighted images shows thick enhancing duramater. Arachnoid granulations are always isointense to CSF on all MR images [5]. Tumours can cause mass affect and its differentiation from the herniation may not difficult. Brain tissue herniation is also a rare cause creating venous sinus filling defect but must be kept in mind. MRI is the best choice to confirm the diagnosis. It is isointense to brain parenchyma on all sequences by magnetic resonance imaging, surrounded by a cerebrospinal fluid rim and is seen to be contiguous with brain tissue on images [6].

In conclusion, it should be considered that brain herniation can be one of the potential cause of filling defects within the dural venous sinuses. Herniation are probably incidental findings, however, it may be associated with headache based our case and other cases defined in literature. There is need to collect more numbers of cases for determination possible relationship.

## Competing Interests

The authors declare that they have no competing interests.
